# Bone formation and resorption markers at 7 years of age: Relations with growth and bone mineralization

**DOI:** 10.1371/journal.pone.0219423

**Published:** 2019-08-22

**Authors:** Teresa Monjardino, Poliana Silva, Joana Amaro, Ofélia Carvalho, João Tiago Guimarães, Ana Cristina Santos, Raquel Lucas

**Affiliations:** 1 EPIUnit—Instituto de Saúde Pública, Universidade do Porto, Porto, Portugal; 2 Serviço de Patologia Clínica, Centro Hospitalar Universitário de São João, Porto, Portugal; 3 Departamento de Biomedicina, Faculdade de Medicina, Universidade do Porto, Porto, Portugal; 4 Departamento de Ciências da Saúde Pública e Forenses e Educação Médica, Faculdade de Medicina, Universidade do Porto, Porto, Portugal; Universite de Nantes, FRANCE

## Abstract

**Purpose:**

We aimed to describe bone formation and resorption markers in generally healthy prepubertal children using total alkaline phosphatase (tALP), osteocalcin (OC) and β-isomerized C-terminal telopeptides of type I collagen (β-CTx) serum concentrations and to estimate markers’ correlations with anthropometric growth (height, weight, body mass index and trajectories of weight gain) as well as bone mineral content (BMC) and areal density (aBMD).

**Methods:**

We assessed 395 7-year-old children from the Generation XXI cohort with tALP, OC and β-CTx concentrations determined from a fasting venous blood sample and BMC/aBMD measured by dual-energy X-ray absorptiometry. Gender-specific reference intervals for tALP, OC and β-CTx in 7-year-old children were established by calculating the 2.5^th^ and 97.5^th^ percentiles. Pearson and partial correlation coefficients (controlling for sex, age, body size and season) between bone markers and growth measures were computed.

**Results:**

tALP increased with height (r_partial_ controlled for sex = 0.26, 95%CI: 0.17, 0.35), was higher in overweight than in healthy weight children, and in children who gained weight above average during infancy. No correlations were found between OC or β-CTx and growth. In girls, OC was slightly correlated with subtotal BMC (r_partial_ = 0.22, 95%CI: 0.08, 0.35), subtotal aBMD (r_partial_ = 0.20, 95%CI: 0.06, 0.33) and lumbar spine aBMD (r_partial_ = 0.23, 95%CI: 0.09, 0.36). tALP and β-CTx were not correlated with any of the DXA-derived bone measures.

**Conclusion:**

This study contributed to the description of bone turnover at 7 years of age and suggested that bone metabolism markers measured in a single point in time have limited ability to describe anthropometric growth and overall bone status in generally healthy prepubertal children.

## Introduction

Serum bone metabolism markers are bone-derived molecules released into circulation as a result of osteoblast or osteoclast activity that can be used to describe bone formation or resorption, respectively [1]. In adults, bone turnover markers reflect the lifelong process of bone remodeling, and have been proposed as independent predictors of bone density and fracture risk [2, 3]. They have also been used to assist the selection of drug treatments for osteoporosis and to monitor the effectiveness of antiresorptive therapies [4, 5].

In children, the meaning of bone metabolism markers is more complex, as they reflect not only background homeostatic remodeling but also two intensive processes that are hallmarks of growth, i.e. bone modeling and linear growth of the skeleton [6]. As such, bone metabolism markers are mainly used in clinical pediatrics for the monitoring of certain chronic conditions that interfere with bone homeostasis and thus with normal skeletal growth and development, including *osteogenesis imperfecta*, juvenile idiopathic arthritis, and chronic kidney disease [7]. Much less is known about the usefulness of bone formation and resorption markers to describe bone development or growth trajectories in the healthy skeleton. From a practical perspective, for instance in large-scale epidemiological studies aiming to assess bone mineralization in population-based samples of children, measurement of serum levels bone formation and resorption could be an interesting alternative to bone mass estimation using dual-energy X-ray absorptiometry (DXA), since the former may demand lower resources, while avoiding radiation exposure [7]. Bone metabolism markers have also the potential to be more sensitive to short-term changes to bone turnover rates [6]. Nevertheless, it is not clear at present whether serum levels of bone metabolism markers add useful information to describe childhood growth, given their high preanalytical variability [8].

Most previous studies exploring the relation between bone metabolism markers and growth have used cross-sectional designs and have described associations in relatively small samples of children, with wide age ranges [9–17]. Additionally, most existing evidence was obtained from convenience or clinical-based samples where distributions of bone markers are not necessarily representative of those in the source population [18, 19]. Therefore, by using data from a population-based sample of prepubertal children of the same age, we aimed to describe bone formation and resorption markers using total alkaline phosphatase (tALP), osteocalcin (OC) and β-isomerized C-terminal telopeptides of type I collagen (β-CTx), and to assess whether those bone metabolism markers are associated with DXA-derived bone properties and anthropometric growth up to seven years of age.

## Materials and methods

We used data from a sub-sample of 395 children, recruited and followed up as part of Generation XXI, a prospective birth cohort assembled in 2005/06 at public maternity units in Porto, Portugal [20, 21].

### Generation XXI cohort assembly and follow-up

All live infants with more than 23 weeks of gestation, born in one of the five public maternity units in the Porto Metropolitan Area, between April 2005 and August 2006, were eligible to participate. Of the invited mothers, 91.4% accepted to participate and their 8647 infants were enrolled in the cohort study. Seven years after birth (April 2012 to April 2014), all Generation XXI participants were invited to a follow-up evaluation and, of the initial cohort, 5849 (67.6%) were interviewed face to face. In each evaluation, we obtained written informed consent from parents or legal guardians and additional oral assent from children at 7 years of age. The study protocol complies with the 1964 Declaration of Helsinki and was approved by the Ethics Committee of *University of Porto Medical School/Hospital de São João*, Porto, Portugal, and registered with the Portuguese Data Protection Authority.

### Study sample

From the 5849 children evaluated at 7 years of age we selected a subsample of 400 participants to measure bone metabolism markers and bioactive molecules: serum 25-hydroxyvitamin D (25(OH)D), intact parathyroid hormone (PTHi), calcium (Ca) and inorganic phosphorus (P_i_). Sample size was calculated based on expected correlations between bone parameters, in order to allow estimating a 0.15 linear correlation coefficient with an 85% power at a 0.05 significance level. These participants were selected based on the following characteristics: 1) to be a singleton full-term baby (more than 36 weeks of gestation); 2) to have a valid whole body DXA scan at 7 years, 3) to have a fasting blood sample collected in the morning with enough volume to perform the intended laboratory analyses. From all participants with the aforementioned characteristics, we randomly selected 200 boys and 200 girls. After samples were sent for analysis, we excluded a further five children as their samples were hemolytic, icteric or lipemic, leaving 395 children for the current analysis (49.9% girls).

Children included in the present study were not different from the remaining cohort regarding sex distribution (49.9% girls vs. 49.0%, p = 0.719), height at 7 years of age (123.3 vs. 123.7 cm, p = 0.146) and weight at 7 years of age (25.9 vs. 26.2 kg, p = 0.176). However, participants included had lower bone mineral content (BMC) (585.0 g in included vs. 604.8 g in excluded participants, p < 0.001) than those excluded.

### Blood sampling and serum determinations

At the 7 years of age follow-up evaluation, we collected blood samples drawn from an antecubital vein, in the morning after overnight fasting. All children were offered local dermal analgesia with lidocaine/prilocaine (EMLA). Blood samples were collected in Vacumed vacuum tubes with gel separator plus clot activator (FL medical, Italy), allowed to clot for 20 minutes at room temperature and centrifuged for 15min at 1500 ×g. Serum was aliquoted and kept frozen at -80°C in the biobank at the University of Porto Medical School until the day of analysis. All analytical measurements presented in this paper were conducted at the clinical pathology laboratory of the *Centro Hospitalar São João*, *EPE* in Porto, Portugal, in June 2014, after being thawed according to standard operating procedures.

Total alkaline phosphatase (tALP) was quantified by a spectrophotometric method based on the conversion of p-nitro-phenylphosphate (pNPP) to p-nitrophenol (pNP) measured at 410/480 nm on an Olympus AU5400 analyzer (Beckman Coulter Inc., USA) (the detection range of the kit, defined by the detection limit and the calibration curve maximum concentration, is 5–1500 units/l, U/l).

Osteocalcin (OC), β-isomerized C-terminal telopeptides of type I collagen (β-crosslaps, β-CTx), and parathyroid hormone measurements were performed by electro-chemiluminescence immunoassays (ECLIA) in a Cobas e411 analyzer (Roche Diagnostics GmbH, Mannheim, Germany) [22]. The Elecsys N-MID Osteocalcin assay (Roche Cobas, Roche Diagnostics, Mannheim, Germany) detects both the stable N-terminal mid-fragment of OC (amino acids 1–43) and intact Osteocalcin (aminoacids 1–49) (detection range: 0.500–300 μg/l). The Elecsys β-crosslaps assay/serum quantifies all degradation fragments of the C-terminal telopeptide region of type I collagen α1 chain that contain the β-isomerized octapeptide EKAHD-β-GGR twice (β-CTx) [23]. This test uses two mouse monoclonal antibodies directed against different regions of the EKAHD-β-GGR octapeptide (detection range: 10–6000 ng/l). Parathyroid hormone was measured as PTHi, a single polypeptide chain containing 84 amino acids. Two different monoclonal antibodies, directed against the aminoacid regions 26–32 (N-terminal) and 37–42 (C-terminal) were used (detection range: 0.127–530 pmol/l).

Serum 25(OH)D concentration was determined using LIAISON 25-OH Vitamin D Total assay (DiaSorin, Saluggia, Italy), a direct competitive chemiluminescence immunoassay which recognizes 25 (OH) vitamin D2 and 25 (OH) vitamin D3 and is fully automated using a Liason platform (detection range: 4.0–150 ng/ml) [24].

Ca and P_i_ were determined using the Olympus AU5400 analyzer. Ca was detected by a colorimetric assay where Calcium ions (Ca^2+^) react with Arsenazo III and produce an intense purple color complex (detection range: 1–5 mmol/l) (Beckman Coulter AU System Calcium Arsenazo, Beckman Coulter). P_i_ was determined by a colorimetric assay were the absorbance at 340/380 nm is directly proportional to the P_i_ level in the sample (Beckman Coulter AU System Inorganic Phosphorus Reagent, Beckman Coulter) (detection range: 0.32–6.40 mmol/l).

All analytical measurements were performed according the manufacturer’s instructions regarding preventive maintenance, function checks, calibration, and quality control of both tests and equipment. All samples tested underwent automated interference analysis for hemolysis, hyperbilirubinemia and lipemia.

### Physical examination

Anthropometry was obtained while the child stood barefoot in light indoor clothing. Weight was measured to the nearest 0.1kg using a digital scale (TANITA, Arlington Heights, IL, USA) and height was measured to the nearest 0.1cm using a wall stadiometer (SECA, Hamburg, Germany). Body mass index (BMI, kg/m^2^) was calculated as the ratio of weight to height squared and BMI-for-age values were classified according to the World Health Organization reference data for BMI z-score into the following categories: normal weight (≤+1 SD) and overweight (>+1 SD, including children with overweight and obesity) [25]. In our sample no children were underweight according to the cut-off value <-2 SD. The definition of weight trajectories in the cohort was based on an extensive body of anthropometric measurements abstracted from the children's health books, recorded in routine care, from birth until the age of 6 years. Four different weight trajectories for both sexes combined were identified through normal mixture modeling for model-based clustering and labelled “normal weight gain”, “weight gain during infancy”, “weight gain during childhood” and “persistent weight gain”, as previously described [26] (Figure A in S1 File).

Whole body DXA scans were performed in a Hologic Discovery QDR 4500W device (Hologic Inc., Bedford, Massachusetts), according to standard manufacturer protocols in light clothing and without metal accessories. Total body less head (subtotal) and lumbar spine (LS) BMC was obtained and areal bone mineral density (aBMD) was expressed as BMC (in g) per projected bone area (in cm^2^) [27]. We performed daily standard quality assurance tests using the spine phantom. Scans were evaluated immediately after the scanning procedure and later validated by a second well-trained radiology technician.

### Data analysis

For the description of reference intervals, according to the C28-A3 International Federation of Clinical Chemistry and Clinical Laboratory and Standards Institute guidelines (C28-A3 CLSI/IFCC guidelines), data were examined for outlier observations using frequency histograms and Dixon outlier range statistic as follows. Estimation of D/R ratio, where D is the absolute difference between the most extreme observation (large or small) and the next largest (or smallest) observation and R is the range of all observations (maximum-minimum) was estimated. If the ratio D/R exceeds 1/3, then the extreme observation is deleted, otherwise all datum is kept [28]. Using this procedure, no extreme values were detected as outliers for any of the bone markers distributions. Concentrations of bone metabolism markers are presented as medians, and the interval limits were determined by calculating the rank numbers for the 2.5^th^ and 97.5^th^ percentiles of the distribution [28]. The season during which blood samples were collected was combined into two categories: summer (April-September) and winter months (October-March).

Two-sample t test, Mann–Whitney test and chi-square test were used to compare boys and girls with regard to bone markers concentrations, age, anthropometrics (height, weight, BMI and trajectories of weight gain), 25(OH)D, PTHi, Ca and P_i_.

Correlations were estimated using parametric statistics (Pearson correlation coefficients) to assess crude relationships among bone metabolism markers and between bone markers and age, height, weight and DXA-derived BMC and aBMD. To account for the effects of age, sex, body size (height and weight) and season on the relationships studied we computed Pearson partial correlations (r_partial_). Correlations below 0.20 were interpreted as very weak, between 0.2 and 0.4 as weak and between 0.4 and 0.6 as moderate. Concentrations of bone markers were compared between BMI groups using Mann–Whitney test and between weight gain trajectories using ANOVA. Mean values of bone markers concentrations, adjusted for sex, age and season were estimated through ANCOVA and comparisons between BMI groups were performed using *nlcom* command in Stata. Mean values of bone markers concentrations according to weight gain trajectories were additionally adjusted for current body size (height and weight at 7 years of age) and comparisons between trajectories were performed using Wald test (*testparm* command in Stata). Post-hoc pairwise comparison of adjusted means between trajectories were performed with the Tukey-Kramer correction for multiple comparison.

### Sensitivity analyses

To assess the impact of vitamin D deficiency on the relationships between bone markers and anthropometrics and BMC/aBMD, we recalculated correlations after excluding children with serum 25(OH)D below 20 ng/ml [29] (n = 153), and compared these estimates with those obtained for the whole sample. Additional sensitivity analyses to assess the impact of hypo- or hypercalcemia and hypo- or hyperphosphatemia were carried out by excluding children with serum Ca or P_i_ concentrations outside reference intervals established by the instrument manufacturer (Ca below 2.2 mmol/l or above 2.7 mmol/l, n = 58; P_i_ below 1.292 mmol/l or above 2.261 mmol/l, n = 3) [30].

All statistical analyses were conducted by using Stata version 11.2 for Windows (Stata Corp. LP, College Station, Texas, USA).

## Results

### Sample characteristics

In our sample of 7-year-old children, reference intervals expressed as 2.5^th^ to 97.5^th^ percentile were 159 to 439 U/l for serum tALP and 470 to 1690 ng/l for β-CTx. Since mean OC concentration was higher in girls than in boys (87.9 versus 82.1 μg/l, respectively, p = 0.003), reference intervals are presented separately by sex: 52.5 to 137.7 μg/l in girls and 50.0 to 129.9 μg/l in boys. Seasonal variation of bone metabolism markers was observed, with increased levels of tALP (p<0.001) and β-CTx (p = 0.003) in children sampled in summer (n = 314) than in those sampled in winter months (n = 81) (Table 1).

**Table 1:** Anthropometrics and serum concentrations of vitamin D, parathyroid hormone, calcium, phosphorus and bone metabolism markers, in girls and boys (n = 395).

	TOTAL	Girls (n = 197)	Boys (n = 198)	Comparison between genders
	**Mean ± SD or Median (P25, P75) or n (%)**			p-value ^a^
**Age (months)**	84.1 (83.6, 84.5)	83.9 (83.7, 84.5)	84.1 (83.6, 84.6)	0.674
**Height (cm)**	123.3 ± 5.1	122.7 ± 4.9	123.8 ± 5.2	0.022
**Weight (kg)**	25.2 (22.6, 28.1)	24.9 (22.6, 28.1)	25.2 (22.7, 28.2)	0.774
**BMI categories (%)** ^b^				
Normal weight	262 (66.3)	127 (64.5)	135 (68.2)	0.719
Overweight	81 (20.5)	42 (21.3)	39 (19.7)	
Obese	52 (13.2)	28 (14.2)	24 (12.1)	
**Growth trajectories (%)**				
Normal weight gain	243 (67.5)	110 (61.1)	133 (73.9)	0.004
Weight gain during infancy	23 (6.4)	9 (5.0)	14 (7.8)	
Weight gain during childhood	54 (15.0)	38 (21.1)	16 (8.9)	
Persistent weight gain	40 (11.1)	23 (12.8)	17 (9.4)	
Missing data for growth trajectory assignment	35	17	18	
**25(OH)D (ng/ml)**				
Total sample	21.8 (17.7, 26.3)	22.0 (17.7, 26.6)	21.7 (17.8, 26.0)	0.756
Collected in summer ^c^	23.0 (19.0, 28.0)	23.3 (19.1, 28.2)	22.6 (18.5, 27.8)	
Collected in winter ^d^	17.2 (14.1, 21.1)	16.2 (12.9, 20.9)	18.0 (14.8, 21.8)	
Vitamin D insufficiency (%) ^e^	180 (45.6)	83 (42.1)	97 (49.0)	
Vitamin D deficiency (%) ^e^	153 (38.7)	81 (41.1)	72 (36.7)	0.583
**PTHi (pmol/l)**	3.24 (2.63, 3.83)	3.28 (2.65, 3.88)	3.20 (2.60, 3.74)	0.608
**Ca (mmol/l)**	2.5 (2.4, 2.6)	2.5 (2.4, 2.6)	2.5 (2.4, 2.6)	0.308
**P**_**i**_ **(mmol/l)**	1.64 ± 0.16	1.63 ± 0.16	1.66 ± 0.16	0.025
	**P50**^**th**^ **(P2.5**^**th**^**—P97.5**^**th**^**)**			
**tALP (U/l)** ^f^				
Total sample	260 (159, 439)	258 (153, 439)	262 (167, 445)	0.820
Collected in summer ^g^	269 (175, 445)	272 (167, 459)	265 (175, 445)	
Collected in winter ^d^	228 (139, 396)	220 (134, 396)	239 (139, 584)	
**OC (μg/l)**				
Total sample	85.2 (50.3, 134.8)	87.9 (52.5, 137.7)	82.1 (50.0, 129.9)	0.003
Collected in summer ^c^	85.6 (51.6, 136.5)	88.3 (52.5, 140.6)	82.2 (51.1, 130.2)	
Collected in winter ^d^	83.6 (50.0, 124.3)	85.2 (59.6, 127.9)	81.6 (48.6, 114.5)	
**β-CTx (ng/l)**				
Total sample	1030 (470, 1690)	1040 (450, 1690)	1005 (510, 1690)	0.258
Collected in summer ^c^	1040 (500, 1690)	1075 (450, 1700)	1025 (530, 1680)	
Collected in winter ^d^	940 (470, 1380)	930 (500, 1320)	940 (440, 1700)	

^a^ Two-sample t-test, Mann–Whitney or chi-square tests as appropriate

^b^ According to World Health Organization reference data for BMI z-score: normal weight (≤1 SD), overweight (>1 SD and ≤2 SD) and obesity (>2 SD) (de Onis, *et al*., Bull World Health Organ, 2007)

^c^ n = 314 (154 Girls + 160 Boys)

^d^ n = 81 (43 Girls + 38 Boys)

^e^ According to the Endocrine Society (United States), vitamin D insufficiency is defined as serum 25(OH)D of 21–29 ng/ml and vitamin D deficiency as serum 25(OH)D below 20 ng/ml (Holick, *et al*., J Clin Endocrinol Metab, 2011)

^f^ n = 394 (197 Girls + 197 Boys)

^g^ n = 313 (154 Girls + 159 Boys)

Abbreviations: BMI, body mass index; 25(OH)D, 25-hydroxyvitamin D; PTHi, intact parathyroid hormone; Ca, calcium, P_i_, inorganic phosphorus; tALP, total alkaline phosphatase; OC, osteocalcin; β-CTx, β-crosslaps

There were no differences between girls and boys in age, weight and BMI distributions. Girls were slightly shorter than boys (122.7 versus 123.8 cm, p = 0.022) had lower serum Pi concentration (1.63 versus 1.66 mmol/l, p = 0.025), and were more likely to have a trajectory of weight gain during childhood. Serum vitamin D level was higher in samples of participants evaluated in summer than in winter months (p<0.001). Vitamin D insufficiency was present in 45.6% children while vitamin D deficiency was present in 38.7% children [29] (Table 1).

### Relationship between bone formation and resorption

In both sexes, positive moderate correlations between OC and β-CTx were found, even after controlling for age, body size and season effects (girls: r_partial_ = 0.40, 95%CI: 0.28, 0.51 and boys: r_partial_ = 0.46, 95%CI: 0.34, 0.56). No relevant correlations were observed between tALP and OC (girls: r_partial_ = 0.02, 95%CI: -0.12 0.16 and boys: r_partial_ = 0.07, 95%CI: -0.07, 0.21) or between tALP and β-CTx (girls: r_partial_ = 0.13, 95%CI: -0.01, 0.27 and boys: r_partial_ = 0.00, 95%CI: -0.14, 0.14) (Table 2).

**Table 2:** Pearson correlation and partial correlation coefficients among bone metabolism markers, in girls and boys (n = 395).

		Girls (n = 197)					
		tALP		OC		β-CTx	
		Coefficient	95%CI	Coefficient	95%CI	Coefficient	95%CI
**Boys (n = 198)**	**tALP**			r = 0.06	-0.08, 0.20	r = 0.18	0.04, 0.31
				r_partial_ = 0.02	-0.12, 0.16	r_partial_ = 0.13	-0.01, 0.27
	**OC**	r = 0.11	-0.03, 0.25			r = 0.40	0.28, 0.51
		r_partial_ = 0.07	-0.07, 0.21			r_partial_ = 0.40	0.28, 0.51
	**β-CTx**	r = 0.06	-0.08, 0.20	r = 0.47	0.36, 0.58		
		r_partial_ = 0.00	-0.14, 0.14	r_partial_ = 0.46	0.34, 0.56		

Abbreviations: tALP, total alkaline phosphatase; OC, osteocalcin; β-CTx, β-crosslaps; r, Pearson correlation coefficient; r_partial_, Pearson partial correlation coefficient (age, body size and season controlled); 95%CI, 95% confidence interval.

### Bone metabolism markers and age and anthropometrics

**Fig 1:**
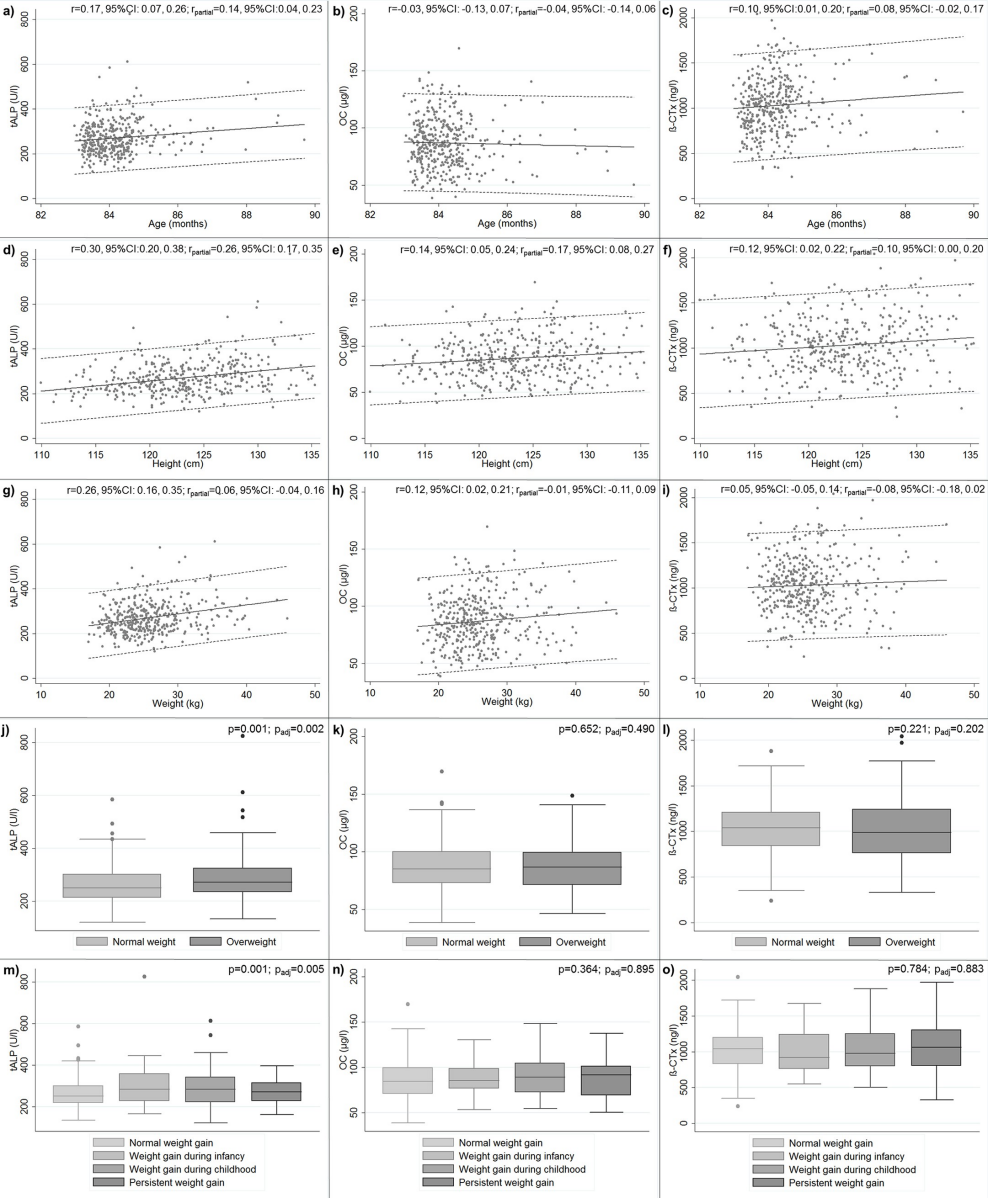
Serum concentrations of bone metabolism markers in relation to age and anthropometrics (n = 395). (a) tALP in relation to age. (b) OC in relation to age. (c) β-CTx in relation to age. (d) tALP in relation to height. (e) OC in relation to height. (f) β-CTx in relation to height. (g) tALP in relation to weight. (h) OC in relation to weight. (i) β-CTx in relation to weight. (j) tALP by BMI groups. (k) OC by BMI groups. (l) β-CTx by BMI groups. (m) tALP by trajectories of weight gain. (n) OC by trajectories of weight gain. (o) β-CTx by trajectories of weight gain. Panels a) to i): the solid line represents the mean and the dotted lines ± 2SD; r, Pearson correlation coefficient, r_partial_, Pearson partial correlation coefficient (correlations between bone metabolism markers and age were controlled for sex and season; between bone metabolism markers and height were controlled for sex, age and season; between bone metabolism markers and weight were controlled for sex, age, height and season). For better data visualization, the participant with 95 months of age is not represented in Panels a) to c). Panels j) to l): p, p-values from the Mann–Whitney test for comparisons between BMI groups; p_adjusted,_ p-values from the *nlcom* postestimation command for comparisons between BMI groups (means of bone metabolism markers were adjusted for sex, age and season). Panel m) to o): p, p-values from ANOVA for comparisons between trajectories of weight gain; p_adjusted,_ p-values from the *testparm* Stata command for comparisons between trajectories of weight gain (means of bone metabolism markers were adjusted for sex, age, current body size and season). Abbreviations: tALP, total alkaline phosphatase; OC, osteocalcin; β-CTx, β-crosslaps; BMI, body mass index, 95% CI, 95% confidence interval.

Correlations between age and tALP and β-CTx were very weak and attenuated after accounting for sex and season effects (r_partial_ = 0.14, 95%CI: 0.04, 0.23 and r_partial_ = 0.08, 95%CI: -0.02, 0.17, respectively) (Fig 1A and Fig 1C).

As regards anthropometrics, tALP was slightly correlated with height (r = 0.30, 95%CI: 0.20, 0.38) (Fig 1D) and weight (r = 0.26, 95%CI: 0.16, 0.35) (Fig 1G) while correlations between height or weight and OC or β-CTx were irrelevant (Fig 1E, Fig 1F, Fig 1H and Fig 1I). After accounting for the effect of sex, age and season, the positive relationship between height and tALP was attenuated (r_partial_ = 0.26, 95%CI: 0.17, 0.35) (Fig 1D). Comparing BMI groups, we observed that overweight children had higher tALP concentration than normal weight children [mean (95% CI) adjusted for sex, age and season: 287 (275, 300) versus 263 (254, 272) U/l, p = 0.002] (Fig 1J). Also for tALP, we observed that, in comparison to children in a “normal weight gain” trajectory [mean (95% CI) adjusted for sex, age and season: 268 (259, 278) U/l)], those in the trajectories of “weight gain during infancy” presented slightly higher serum concentrations [308 (278, 339) U/l, p = 0.018] (Fig 1M). For OC and β-CTx, we observed no differences in serum concentrations by BMI groups or by trajectories of weight gain (Fig 1K, Fig 1L, Fig 1N and Fig 1O).

### Bone metabolism markers and DXA-derived bone measures

Overall, Table 3 shows that correlations between bone metabolism markers and DXA-derived BMC and aBMD were positive but modest in magnitude. Crude correlations with BMC and aBMD were slightly stronger for tALP than for OC or β-CTx. Serum OC was slightly stronger correlated with BMC/aBMD in girls than in boys. However, after computing partial correlations accounting for age, body size and season effects, tALP was no longer correlated with bone mass, neither in girls nor in boys. In girls only, weak positive correlations between OC and subtotal BMC (r_partial_ = 0.22, 95%CI: 0.08, 0.35), subtotal aBMD (r_partial_ = 0.20, 95%CI: 0.06, 0.33) and lumbar spine aBMD (r_partial_ = 0.23, 95%CI: 0.09, 0.36) were observed. β-CTx was not correlated with any of the DXA-derived bone measures.

**Table 3:** Pearson correlation and partial correlation coefficients between bone metabolism markers and bone mineral content and density, in girls and boys (n = 395).

		Subtotal BMC		Lumbar spine BMC		Subtotal aBMD		Lumbar spine aBMD	
		Coefficient	95%CI	Coefficient	95%CI	Coefficient	95%CI	Coefficient	95%CI
**tALP**	**Girls**	r = 0.25	0.12, 0.38	r = 0.18	0.05, 0.32	r = 0.24	0.11, 0.37	r = 0.19	0.06, 0.33
		r_partial_ = 0.02	-0.12, 0.16	r_partial_ = 0.08	-0.06, 0.22	r_partial_ = 0.02	-0.12, 0.16	r_partial_ = 0.09	-0.05, 0.23
	**Boys**	r = 0.24	0.11, 0.37	r = 0.22	0.08, 0.35	r = 0.22	0.08, 0.35	r = 0.20	0.06, 0.33
		r_partial_ = 0.11	-0.03, 0.25	r_partial_ = 0.13	-0.01, 0.27	r_partial_ = 0.10	-0.04, 0.23	r_partial_ = 0.13	-0.01, 0.26
**OC**	**Girls**	r = 0.23	0.09, 0.36	r = 0.14	0.00, 0.28	r = 0.20	0.06, 0.33	r = 0.26	0.12, 0.38
		r_partial_ = 0.22	0.08, 0.35	r_partial_ = 0.10	-0.04, 0.23	r_partial_ = 0.20	0.06, 0.33	r_partial_ = 0.23	0.09, 0.36
	**Boys**	r = 0.19	0.05, 0.32	r = 0.06	-0.08, 0.20	r = 0.14	0.00, 0.28	r = 0.13	-0.01, 0.27
		r_partial_ = 0.08	-0.06, 0.21	r_partial_ = -0.07	-0.21, 0.07	r_partial_ = 0.04	-0.10, 0.18	r_partial_ = 0.06	-0.08, 0.19
**β-CTx**	**Girls**	r = 0.07	-0.07, 0.21	r = 0.08	-0.06, 0.22	r = 0.01	-0.13, 0.15	r = 0.04	-0.10, 0.18
		r_partial_ = 0.11	-0.03, 0.25	r_partial_ = 0.04	-0.10, 0.18	r_partial_ = 0.03	-0.11, 0.17	r_partial_ = 0.05	-0.09, 0.19
	**Boys**	r = 0.10	-0.04, 0.24	r = 0.07	-0.07, 0.21	r = 0.06	-0.08, 0.20	r = 0.09	-0.05, 0.22
		r_partial_ = 0.04	-0.10, 0.18	r_partial_ = -0.02	-0.16, 0.12	r_partial_ = -0.00	-0.14, 0.14	r_partial_ = 0.06	-0.08, 0.19

Abreviations: tALP, total alkaline phosphatase; OC, osteocalcin; β-CTx, β-crosslaps; BMC, bone mineral content; aBMD, areal bone mineral density; r, Pearson correlation coefficient; r_partial_, Pearson partial correlation coefficient (age, body size and season controlled); 95% CI, 95% confidence interval.

### Sensitivity analyses

After excluding children with vitamin D deficiency (n = 153), with hypo- and hypercalcemia (n = 58) or hypo- and hyperphosphatemia (n = 3), one group at a time, correlation coefficients between bone metabolism markers and anthropometrics and DXA-derived bone measures remained similar to those obtained for the whole sample of children before exclusion of these subjects.

## Discussion

In this study, we described reference intervals for tALP, OC and β-CTx in a population-based sample of 7-year-old children. We found a moderate correlation between serum concentrations of bone-specific metabolism markers OC and β-CTx, likely representing the dynamic nature of bone turnover. The non-specific bone marker tALP was slightly more correlated than OC or β-CTx with bone mass and anthropometric variables, probably reflecting the overall trajectory of anthropometric growth up to the time of measurement.

There has been growing interest in the quantification of bone metabolism markers in the clinical setting as they may provide a dynamic, short-term measure of skeletal status, which is not captured by bone physical properties alone [2]. So far, no single individual parameter has fulfilled all the criteria for an ideal marker of bone formation or resorption [6]. The clinical utility of bone markers is challenged by high intraindividual variation, lack of specificity for bone tissue, release during different anabolic and catabolic processes and influence of non-skeletal processes on circulating levels [31]. Also, no single marker is sufficiently precise to be used for prognostic purposes, as concentration changes of candidate molecules are neither site- nor disease-specific [6]. An additional challenge is posed in the interpretation of bone metabolism markers as a prognostic tool, in populations of children and adolescents, because it is impossible to distinguish treatment-induced changes from the physiological age-related decline in these bone markers [31]. Furthermore, some bone metabolism markers, such as OC, may reflect, at the same time, both bone formation and resorption processes [31]. Because there is no specific bone marker to assess bone modeling, remodeling or epiphyseal growth, concentrations of markers in children represent the combined effects of these different biological processes [6]. This means that equal serum levels of bone metabolism markers can be found both in children with high bone remodeling and low rate of growth as well as in normally growing children [32].

In addition to limitations in inferring the biological significance of bone metabolism markers, the definition of pediatric reference intervals is limited by their substantial pre-analytical variability and by heterogeneity in analytical methods [8]. Pre-analytical sources of variability include uncontrollable factors (such as sex, age, pubertal development, growth velocity, ethnicity, physical activity, nutritional status, or pathological conditions (e.g. diabetes, liver diseases, growth hormone deficiency, vitamin D deficiency, or recent fracture) and drugs), and controllable factors such as circadian rhythm, seasonal variation or fasting status [8]. The main sources of undesired pre analytical variability of tALP are related to age, pubertal status and sex, as well as lack of bone specificity [32]. Regarding OC, pre-analytical variability is mostly related to age and circadian rhythm [8]. β-CTx also exhibits significant circadian rhythm and is influenced by food intake, as well as age [8]. Analytical variability further contributes to variation of reference intervals, due to lack of standardization and harmonization of quantification methods and to inter-laboratory variation, even when the same method is used [33]. Attempts to establish reference intervals have also been limited by the small sample sizes of most previous investigations, which have also been frequently conducted in hospital settings [33]. Indeed, reference values obtained from facility-based samples may not be applicable to the general pediatric population as intervals in the former are generally wider [34].

To account for those limitations, our approach in the present study was to use data from a comparatively large population-based sample of children of the same age, whose samples were drawn in the morning, after an overnight fast. Nevertheless, our reported reference values are directly applicable only to children of the same age whose samples were tested using laboratory methods similar to ours [28], as supported by our comparison of the present results with previous descriptions (Tables A-C in S1 File). Regarding 25(OH)D, PTHi, Ca and P_i_ we found that concentrations in our sample were within published reference intervals although no previous studies have reported sex differences in serum concentration of P_i_ [35, 36]. However, our findings on the relationships between bone metabolism markers and anthropometrics or DXA-derived bone measures are more dependent on the relative positions of children in the distribution of each marker and less on absolute value of marker concentrations, which makes for safer generalization of the magnitude of associations to other contexts.

We found a positive correlation between OC and β-CTx, in agreement with findings in other samples of prepubertal children [17] but in contrast with one study including 5 to 10 year-old children [14]. Positive relationships between bone-specific formation and resorption markers are expected, and reflect the nature of bone turnover as a tightly regulated dynamic mechanism of formation and resorption [6]. Our absence of correlation between tALP and OC corroborates previous findings [37] but contradicts more recent results that showed direct correlations among formation markers [13, 38]. The recent hypothesis that OC is a marker of bone turnover as a whole, reflecting not only bone formation but also resorption, may explain its less consistent associations with other formation markers [39]. Our results also differed from previous findings that disclosed a positive relationship between ALP and β-CTx. However, other studies have assessed bone-specific ALP rather than total ALP [38]. In addition, since previous studies have looked at wider age ranges, results are not straightforwardly comparable to ours in children of the same age.

Overall, weak correlations among bone markers are probably due to the coexistence of different biological processes at different anatomical regions and bone surfaces during skeletal growth. In addition, markers are released during different stages of bone formation and resorption, and have different elimination pathways and serum half-lives, which may affect their relations at different time points during growth [6].

Our results were indicative of a weak positive effect of age on tALP concentrations, which corroborates previous findings of an age-related increase in tALP, expected to peak at approximately 10–12 years of age in girls and 13–15 years in boys, and detectable around 7 years of age [35, 36, 40–43]. At this age, tALP was slightly correlated with height. Also, higher concentrations were observed in overweight when compared to healthy weight participants, and in children who gained weight above-average during infancy, in comparison to those in an average weight gain trajectory. These modest positive relationships between tALP and anthropometric growth, which were not apparent for the bone-specific markers OC and β-CTx, suggest that tALP is a more specific marker of the child’s overall growth trajectory up to the time of measurement. Positive correlations between bone-specific ALP and anthropometric measures have been reported elsewhere [38] while others shown no effect of BMI on tALP values [44]. Our results did not support previously described positive correlations between OC and height and weight [10, 14], between CTX and weight [14] and height [13, 38] or the finding of decreased OC values in obese when compared to healthy weight children [11, 12]. However, in accordance to our findings, a number of other studies have contributed with evidence of a lack of association between height, weight or BMI and OC [9, 10, 13, 14, 39, 44] and height or BMI and β-CTx [44]. Increased formation markers in taller and heavier children are likely reflecting greater growth velocity, inducing increased bone formation in response to greater mechanical strain [13, 32]. Accordingly, CTX may also correlate with height since increased periosteal modeling also implies removal of bone at the endocortical surface [45]. To the best of our knowledge, our study is the first to estimate relationships between bone markers and growth trajectories from birth. Given the lack of relationships between OC or β-CTx and trajectories of weight gain we believe that, contrarily to dimensions of skeletal growth, such as BMC, that are clearly shaped by the overall weight trajectory, markers of bone metabolism are not related to overall weight trajectories [46]. The lack of associations between OC or β-CTx and the remaining anthropometric growth indices supports this assertion.

Bone metabolism markers were also only weakly correlated with DXA-derived BMC and aBMD. Additional adjustments for age, body size and season attenuated those relationships only for OC and only in girls. This suggests that measurements of bone metabolism markers at a single point in time do not reflect bone mineralization status in the general prepubertal population and are unlikely to be helpful for monitoring the status of bone mineral accrual in non-clinical settings. These findings are consistent with the premise that bone markers reflect instantaneous metabolic activity and do not directly translate physical dimensions that result from the cumulative process of bone gain throughout several years [6]. In prepubertal children, some previous studies have also found no relationship between tALP, OC or β-CTx and whole body (WB) and LS BMC or BMD [14, 15, 35]. Other studies disclosed, however, consistent positive relationships between tALP and OC and WB and LS BMC, in boys [16] and between OC and CTX and WB and LS BMC/BMD, in both sexes [15, 17]. In older pubertal children, inverse correlations between serum OC or CTX and BMD were disclosed [9, 15], which is expected because, during puberty, bone turnover decreases with advancing sexual maturation while, at the same time, bone mass accrual continues up to peak bone mass [7]. Some authors have also described site-specific associations between bone metabolism markers and bone mass during puberty, disclosing stronger relationships in anatomical sites with more trabecular bone (e.g. LS BMC) than with cortical bone (FN BMC), possibly due to higher metabolic activity of trabecular when compared to cortical bone [47]. In our study, however, bone markers were not correlated with either cortical or trabecular bone when we computed correlations between bone markers and BMC and aBMD at the LS and lower limbs (Table D in S1 File).

This study extends previous evidence by assessing the usefulness of bone markers to describe growth in a population-based sample of children with longitudinal anthropometric data. In particular, we were able to investigate for the first time the relationships of bone markers with weight gain trajectories since birth. We also quantified bone metabolism markers at the same chronological age for all participants. Furthermore, blood samples were all collected in the morning after overnight fasting to avoid the variability associated to the circadian rhythm. In addition, reference intervals were described according the statistical approach recommended in the C28-A3 CLSI/IFCC guidelines [28]. However, we should acknowledge a limitation in our use of tALP instead of bone-specific ALP. Serum variations of tALP are not as accurate as bone-specific ALP to detect subtle changes in bone formation [6]. Nevertheless, when liver ALP is stable and remains within normal values, tALP represents a valid marker of bone turnover [31].

In the future, it will be interesting to investigate time changes in bone markers and whether those are associated with changes in bone mass and density across the lifespan. In particular, it might be important to explore how puberty relates to bone formation and resorption. Although we were not able to assess sexual development in this evaluation, we expect that the vast majority of children from the general population were prepubertal at 7 years of age, as observed in previous population-based samples of the same age [48, 49].

In conclusion, we found weak or negligible associations of bone metabolism markers with different indices of growth, suggesting limited ability of those markers to predict bone status and overall growth in 7-year-old children from the general population.

## Supporting information

S1 Dataset The minimal anonymised dataset including all variables used in statistical analyses here reported. (SAV)

S1 File Reference intervals for serum total alkaline phosphatase concentrations (U/l) (Table A) for serum osteocalcin concentrations (μg/l) (Table B) and for serum β-crosslaps concentrations (ng/l) (Table C). Figure A. Weight trajectories in the Generation XXI cohort. Table D. Pearson correlation and partial correlation coefficients between bone metabolism markers and bone mineral content and density in lower limbs, in girls and boys (n = 395).
(DOCX)
