# Supplementary material for: Bone formation and resorption markers at 7 years of age: Relations with growth and bone mineralization
Source: PLoS One. 2019 Aug 22;14(8):e0219423. doi: 10.1371/journal.pone.0219423 (PMC6705799; doi:10.1371/journal.pone.0219423)
Supplement: S1 File — Reference intervals for serum total alkaline phosphatase concentrations (U/l) (Table A) for serum osteocalcin concentrations (μg/l) (Table B) and for serum β-crosslaps concentrations (ng/l) (Table C). Figure A. Weight trajectories in the Generation XXI cohort. Table D. Pearson correlation and partial correlation coefficients between bone metabolism markers and bone mineral content and density in lower limbs, in girls and boys (n = 395). (DOCX) [file pone.0219423.s002.docx]

**Table A.** Reference intervals for serum total alkaline phosphatase concentrations (U/l)

| First author, year | Age (years) | N | Analytic platform | P50 (P2.5, P97.5) |
| --- | --- | --- | --- | --- |
| Both sexes | | | | |
| *Generation XXI, 2019* | 7 | 393 | Beckman Coulter Olympus AU5400 analyzer | 260 (159, 439) |
| *Wanjian, 2017 (1)* | 6-12 | 175 | Beckman Coulter AU5800 analyzer | (48.8, 445.9) |
| *Abou El Hassan, 2015 (2)* | 1-10 | 391 | Beckman Coulter AU analyzer | (160, 381) |
| *Buchanan, 2015 (3)* | 5-13 | 121 | Cobas Integra 400 plus analyzer | 278 (IC95%: 174, 460) |
| *Ridefelt, 2014 (4)* | 2-8 | 234 | Abbott Architect ci8200 analyzer | (111, 277) |
| *Hilsted, 2013 (5)* | 7-8 | 228 | Roche Modular Analytics P/ISE-System analyzer | (154, 358) |
| Girls | | | | |
| *Generation XXI, 2019* | 7 | 197 | Beckman Coulter Olympus AU5400 analyzer | 258 (153, 439) |
| *Zierk, 2017 (6)* | 7 | 3785 | Roche Cobas analyzer | 217 (127, 345) |
| *Cho, 2014 (7)* | 6-12 | 988 | Hitachi 7600 analyzer | (123, 330) |
| *Hilsted, 2013 (5)* | 7-8 | 135 | Roche Modular Analytics P/ISE-System analyzer | (157, 365) |
| *Huang, 2011 (8)* | 6-11 | 36 | Roche P-modular analyzer | (157, 359) |
| Boys | | | | |
| *Generation XXI, 2019* | 7 | 196 | Beckman Coulter Olympus AU5400 analyzer | 262 (167, 445) |
| *Zierk, 2017 (6)* | 7 | 4237 | Roche Cobas analyzer | 208 (125, 323) |
| *Cho, 2014 (7)* | 6-12 | 1094 | Hitachi 7600 analyzer | (118, 341) |
| *Hilsted, 2013 (5)* | 7-8 | 93 | Roche Modular Analytics P/ISE-System analyzer | (149, 345) |
| *Huang, 2011 (8)* | 6-15 | 75 | Roche P-modular analyzer | (136, 414) |

**Table B.** Reference intervals for serum osteocalcin concentrations (µg/l)

| First author, year | Age (years) | N | Assay/Analytic platform | P50 (P2.5, P97.5) | |
| --- | --- | --- | --- | --- | --- |
| Both sexes | | | | |  |
| *Generation XXI, 2019* | 7 | 395 | Roche Cobas e411 analyzer | 85.2 (50.3, 134.8) | |
| *Bayer, 2014 (9)* | 7.1 - 8 | 15 | Roche Elecsys analyzer | (12.5, 232.5) | |
| *Manjon, 2004 (10)* | 4-10 | 25 | Metra Biosystems NovoCalcin assay | Mean (IC95%:): 15.128 (12.30, 17.95) | |
| Girls | | | | |  |
| *Generation XXI, 2019* | 7 | 197 | Roche Cobas e411 analyzer | 87.9 (52.5, 137.7) | |
| *Huang, 2011 (8)* | 6-10 | 33 | Roche P-modular analyzer | (61.4, 136.2) | |
| *Rauchenzauner, 2007 (11)* | 7 | 32 | Active Human Osteocalcin IRMA | 19.96 (P3, P97: 7.90, 50.44) | |
| *Vietri, 2006 (12)* | 6.1-8 | 207 | DiaSorin LIASON analyzer | Mean: 69.06 (31.46, 92.10) | |
| *Seydewitz, 2001 (13)* | 6-8 | 25 | Immulite immunoanalyzer | 31.3 (P5, P95: 18.6, 62.0) | |
| *Cioffi, 1997 (14)* | 7 | 42 | CIS Bio International IRMA | 72.6 (P25, P75: 53, 97) | |
| Boys | | | | |  |
| *Generation XXI, 2019* | 7 | 198 | Roche Cobas e411 analyzer | 82.1 (50, 129.9) | |
| *Huang, 2011 (8)* | 6-9 | 30 | Roche P-modular | (56.5, 152.1) | |
| *Rauchenzauner, 2007 (11)* | 7 | 32 | Active Human Osteocalcin IRMA | 19.54 (P3, P97: 8.12, 47.00) | |
| *Vietri, 2006 (12)* | 6.1-8 | 209 | DiaSorin LIASON analyzer | Mean: 65.6 (27.48, 98.36) | |
| *Seydewitz, 2001 (13)* | 6-8 | 34 | Immulite immunoanalyzer | 31.6 (P5, P95: 10.6, 50.5) | |
| *Cioffi, 1997 (14)* | 7 | 51 | CIS Bio International IRMA | 66.8 (P25, P75: 49, 104) | |

**Table C.** Reference intervals for serum β-crosslaps concentrations (ng/l)

| First author, year | Age (years) | N | Assay/Analytic platform | P50 (P2.5, P97.5) |
| --- | --- | --- | --- | --- |
| Both sexes | | | | |
| *Generation XXI, 2019* | 7 | 395 | Roche Cobas e411 analyzer | 1030 (470, 1690) |
| *Crofton, 2002 (15)* | 1-9 | 124 | Osteometer Biotech ELISA | 352 (95%CI: 146, 818) |
| Girls | | | | |
| *Generation XXI, 2019* | 7 | 197 | Roche Cobas e411 analyzer | 1040 (450, 1690) |
| *De Melo, 2018 (16)* | 6-9 | 23 | Roche Cobas e170 analyzer | (565, 1570) |
| *Gennai, 2016 (17)* | 7 | NA | Technogenetics-Bouty DSX system | 2000 (P10, P90: 500, 3000) ^a^ |
| *Herrmann, 2014 (18)* | 7.5 | 186 | Roche ECLIA Modular E17 | 1260 (P3, P97: 750, 1770) ^b^ |
| *Alberti, 2011 (19)* | 7-8 | NA | Immunodiagnosticsystems ELISA | 2078 (1413, 3410) |
| *Huang, 2011 (8)* | 6-10 | 33 | Roche P-modular | (820, 2060) |
| *Rauchenzauner, 2007 (11)* | 7 | 32 | Osteometer Biotech One Step ELISA | 1836 (P3, P97: 897, 3109) |
| Boys | | | | |
| *Generation XXI, 2019* | 7 | 198 | Roche Cobas e411 analyzer | 1005 (510, 1690) |
| *De Melo, 2018 (16)* | 6-9 | 28 | Roche Cobas e170 analyzer | (509, 1697) |
| *Gennai, 2016 (17)* | 7 | NA | Technogenetics-Bouty DSX system | 1500 (P10, P90: 300, 3300) ^a^ |
| *Herrmann, 2014 (18)* | 7.0-7.9 | 198 | Roche ECLIA Modular E17 | 1230 (P3, P97: 750, 1710) ^b^ |
| *Alberti, 2011 (19)* | 7-8 | NA | Immunodiagnosticsystems ELISA | 2151 (1285, 2902) |
| *Huang, 2011 (8)* | 6-9 | 29 | Roche P-modular | (1050, 2380) |
| *Rauchenzauner, 2007 (11)* | 7 | 32 | Osteometer Biotech One Step ELISA | 1637 (P3, P97: 787, 2794) |

^a^ Abstracted from visual inspection of the reference curves

^b^ Assuming a mean height of 126 cm in girls and 127 cm in boys

**Figure A.** Weight trajectories in the Generation XXI cohort.


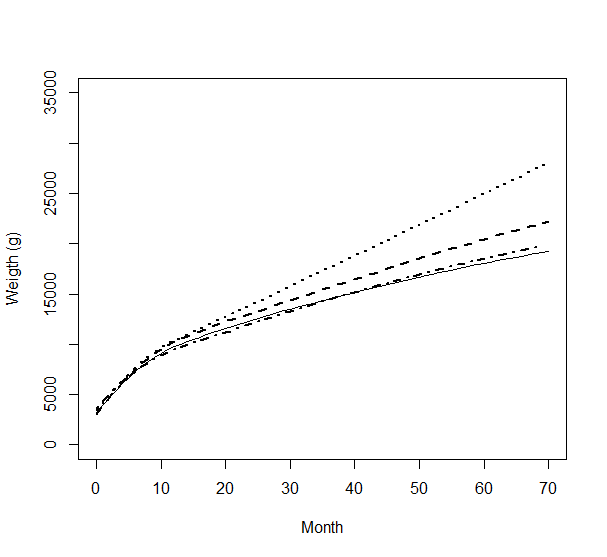


Solid line, “normal weight gain” trajectory; dashed-dotted line, “weight gain during infancy” trajectory; dashed line, “weight gain during childhood” trajectory; dotted line, “persistent weight gain” trajectory.

**Table D.** Pearson correlation and partial correlation coefficients between bone metabolism markers and bone mineral content and density in lower limbs, in girls and boys (n=395).

|  |  | **Lower limbs BMC** | | **Lower limbs aBMD** | |
| --- | --- | --- | --- | --- | --- |
|  |  | Coefficient | 95%CI | Coefficient | 95%CI |
| **tALP** | **Girls** | r=0.26 | 0.12, 0.38 | r=0.25 | 0.12, 0.38 |
|  |  | r_partial_=0.04 | -0.11, 0.17 | r_partial_=0.04 | -0.10, 0.18 |
|  | **Boys** | r= 0.26 | 0.13, 0.39 | r=0.22 | 0.08, 0.35 |
|  |  | r_partial_=0.15 | 0.01,0.28 | r_partial_=0.09 | -0.05, 0.23 |
| **OC** | **Girls** | r=0.25 | 0.11, 0.38 | r=0.18 | 0.04, 0.31 |
|  |  | r_partial_=0.25 | 0.12, 0.38 | r_partial_=0.16 | 0.02, 0.30 |
|  | **Boys** | r=0.18 | 0.04, 0.31 | r=0.14 | 0.00, 0.28 |
|  |  | r_partial_=0.06 | -0.08, 0.20 | r_partial_=0.05 | -0.08, 0.19 |
| **β-CTx** | **Girls** | r=0.08 | -0.06, 0.22 | r=0.01 | -0.13, 0.15 |
|  |  | r_partial_=0.11 | -0.03, 0.25 | r_partial_=0.04 | -0.10, 0.18 |
|  | **Boys** | r=0.09 | -0.05, 0.22 | r=0.06 | -0.08, 0.20 |
|  |  | r_partial_=0.00 | -0.14, 0.14 | r_partial_=0.00 | -0.14, 0.14 |

Abreviations: tALP, total alkaline phosphatase; OC, osteocalcin; β-CTx, β-crosslaps; BMC, bone mineral content; aBMD, areal bone mineral density; r, Pearson correlation coefficient, r_partial_, Pearson partial correlation coefficient (age, body size and season controlled), 95%CI, 95% confidence interval.

**References**

1. Wanjian G, Jie H, Liang G, Cheng W, Tian X, Jianjiang S, et al. Establishment of Reference Interval for Alkaline Phosphatase in Healthy Children of Various Ethnicities, Aged 0-12 Years. Laboratory medicine. 2017;48(2):166-71.

2. Abou El Hassan M, Stoianov A, Araujo PA, Sadeghieh T, Chan MK, Chen Y, et al. CLSI-based transference of CALIPER pediatric reference intervals to Beckman Coulter AU biochemical assays. Clinical biochemistry. 2015;48(16-17):1151-9.

3. Buchanan AM, Fiorillo SP, Omondi MW, Cunningham CK, Crump JA. Establishment of biochemistry reference values for healthy Tanzanian infants, children and adolescents in Kilimanjaro Region. Tropical medicine & international health : TM & IH. 2015;20(11):1569-77.

4. Ridefelt P, Gustafsson J, Aldrimer M, Hellberg D. Alkaline phosphatase in healthy children: reference intervals and prevalence of elevated levels. Hormone research in paediatrics. 2014;82(6):399-404.

5. Hilsted L, Rustad P, Aksglaede L, Sorensen K, Juul A. Recommended Nordic paediatric reference intervals for 21 common biochemical properties. Scandinavian journal of clinical and laboratory investigation. 2013;73(1):1-9.

6. Zierk J, Arzideh F, Haeckel R, Cario H, Fruhwald MC, Gross HJ, et al. Pediatric reference intervals for alkaline phosphatase. Clinical chemistry and laboratory medicine. 2017;55(1):102-10.

7. Cho SM, Lee SG, Kim HS, Kim JH. Establishing pediatric reference intervals for 13 biochemical analytes derived from normal subjects in a pediatric endocrinology clinic in Korea. Clinical biochemistry. 2014;47(18):268-71.

8. Huang Y, Eapen E, Steele S, Grey V. Establishment of reference intervals for bone markers in children and adolescents. Clinical biochemistry. 2011;44(10-11):771-8.

9. Bayer M. Reference values of osteocalcin and procollagen type I N-propeptide plasma levels in a healthy Central European population aged 0-18 years. Osteoporosis international : a journal established as result of cooperation between the European Foundation for Osteoporosis and the National Osteoporosis Foundation of the USA. 2014;25(2):729-36.

10. Manjon Llorente G, Fernandez-Espuelas C, Gonzalez Lopez JM, Ruiz-Echarri MP, Baldellou Vazquez A. [Normal values of bone turnover markers in childhood]. Anales de pediatria (Barcelona, Spain : 2003). 2004;60(4):330-6.

11. Rauchenzauner M, Schmid A, Heinz-Erian P, Kapelari K, Falkensammer G, Griesmacher A, et al. Sex- and age-specific reference curves for serum markers of bone turnover in healthy children from 2 months to 18 years. The Journal of clinical endocrinology and metabolism. 2007;92(2):443-9.

12. Vietri MT, Sessa M, Pilla P, Misso M, Di Troia D, Sorriento A, et al. Serum osteocalcin and parathyroid hormone in healthy children assessed with two new automated assays. Journal of pediatric endocrinology & metabolism : JPEM. 2006;19(12):1413-9.

13. Seydewitz HH, Henschen M, Kuhnel W, Brandis M. Pediatric reference ranges for osteocalcin measured by the Immulite analyzer. Clinical chemistry and laboratory medicine. 2001;39(10):980-2.

14. Cioffi M, Molinari AM, Gazzerro P, Di Finizio B, Fratta M, Deufemia A, et al. Serum osteocalcin in 1634 healthy children. Clinical chemistry. 1997;43(3):543-5.

15. Crofton PM, Evans N, Taylor MR, Holland CV. Serum CrossLaps: pediatric reference intervals from birth to 19 years of age. Clinical chemistry. 2002;48(4):671-3.

16. de Melo VCP, Ferreira PRS, Ricardi LO, Batista MC, Franca CN, Ferreira C. Definition of reference ranges for beta-isomerized carboxy-terminal telopeptide collagen type I for children and adolescents. Journal of pediatric endocrinology & metabolism : JPEM. 2018.

17. Gennai I, Di Iorgi N, Reggiardo G, Gatti C, Bertelli E, Allegri AE, et al. Age- and sex-matched reference curves for serum collagen type I C-telopeptides and bone alkaline phosphatase in children and adolescents: An alternative multivariate statistical analysis approach. Clinical biochemistry. 2016;49(10-11):802-7.

18. Herrmann D, Intemann T, Lauria F, Marild S, Molnar D, Moreno LA, et al. Reference values of bone stiffness index and C-terminal telopeptide in healthy European children. International journal of obesity (2005). 2014;38 Suppl 2:S76-85.

19. Alberti C, Chevenne D, Mercat I, Josserand E, Armoogum-Boizeau P, Tichet J, et al. Serum concentrations of insulin-like growth factor (IGF)-1 and IGF binding protein-3 (IGFBP-3), IGF-1/IGFBP-3 ratio, and markers of bone turnover: reference values for French children and adolescents and z-score comparability with other references. Clinical chemistry. 2011;57(10):1424-35.
